# Factors Associated with Postpartum Depressive Symptoms in Community of Central Nepal

**DOI:** 10.1155/2020/8305304

**Published:** 2020-04-04

**Authors:** Pratima Dawadi, Aarati Sharma Bhatta, Jayalaxmi Shakya

**Affiliations:** School of Nursing, Chitwan Medical College, Bharatpur, Chitwan, Nepal

## Abstract

**Background:**

Pregnancy and postpartum are considered as high risk periods for the emergence of psychiatric disorder. Although postpartum depressive symptoms have been associated with tragic outcome, such as maternal suicide and infanticide, it is a neglected area of mental health care in developing countries. This study was conducted to find the prevalence and factors associated with postpartum depressive symptoms.

**Method:**

A community-based cross-sectional research design was carried out after selecting the three wards of Bharatpur submetropolitan by nonprobability purposive sampling method. A total of 160 mothers in their 1 month to 12 months of postpartum period were interviewed through semistructured interview schedule and Edinburgh Postnatal Depression Scale (EPDS). Collected data were entered in Epi, data 3.1, and was exported into IBM SPSS 20 version.

**Results:**

The prevalence of depressive symptoms among postpartum mothers was 27.5%. The multivariate analysis identified two factors significantly associated with postpartum depression including respondents who had education level of ≤10 class (odds ratio [AOR] = 3.25, *P* = 0.03, confidence interval [CI] = 1.10 − 9.58), chronic disease in their family (odds ratio [AOR] = 3.25, *P* = 0.01, confidence interval [CI] = 1.19 − 8.16).

**Conclusion:**

More than one out of four mothers is suffering from depressive symptoms. The major factors associated with postpartum depressive symptoms are education of respondents and chronic disease in the family. Screening and timely management of depressive symptoms should be incorporated in routine maternal care so as to enhance maternal and child health. Likewise, concerned authority should plan and organize awareness-raising programs and provide attractive package to attract the female population for higher education.

## 1. Background

Postpartum depression (PPD) is a mood disorder to describe the various disorders such as anxiety, loss of enjoyment, sadness, and fatigue which may have serious adverse long-term effects on both the children and mothers [1]. It is common for women to experience the “baby blues” feeling stressed, sad, anxious, lonely, tired, or weepy following their baby's birth, some women, up to 1 in 7, experience postpartum depression (PPD [2].

About 7% of the global burden of diseases among women is attributed to mental health problems, especially among women of reproductive age. Postpartum nonpsychotic depression is the most common complication of childbearing affecting approximately 10-15% of women and as such represents a considerable public health problem affecting women and their families [3] which ultimately affects progress towards the achievement of several Sustainable Development Goals (SDGs) by 2030.

Many researchers who used the Edinburgh postnatal depression scale have been used the term post-partum depression and post-partum depressive symptoms interchangeably. The concept of depression was used widely to mean depressive symptoms, even though depression criteria are defined in Diagnostic and Statistical Manual of Mental Disorder [4]. The Edinburgh Postpartum Depression Scale has been translated from the original English version to a number of languages and has been validated and used successfully in detecting postnatal depression in many countries [5].

A meta-analysis of 59 studies from North America, Europe, Australia and Japan found an average prevalence rate of postpartum depressive symptoms of 13% [6]. Prevalence rates of postpartum depressive symptoms among postpartum women in developing countries India and Nepal vary from 11 to 30% [7, 8].

Postpartum depressive symptoms have been associated with tragic outcome, such as maternal suicide and infanticide; it is a neglected area of mental health care in developing countries. Hence, the aim of this study is to determine the prevalence and various factors associated with depressive symptoms among postpartum mothers.

## 2. Methods

### 2.1. Study Setting

The study was conducted in three selected wards of Bharatpur metropolitan. Bharatpur is a city situated in the central-southern part of Nepal. Study population was women in their 4 weeks to 1 year of postpartum period [2].

### 2.2. Sampling Procedure

First of all, nonprobability purposive sampling technique was used to select the three different wards of Bharatpur submetropolitan of central Nepal from November 2017 to May 2018. Total list of postpartum mothers who met the inclusion criteria of the study was taken from the immunization record register of Shivanagar Primary Health Center (PHC). There was a record of 160 postpartum mothers and all recorded number of mothers was taken as a sample.

### 2.3. Research Instrument

Semistructured interview schedule was developed by researcher herself after reviewing of related literature for sociodemographic characteristic and factors associated with depressive symptoms. Researcher also used the standard tool (Edinburgh Postpartum Depression Scale) to measure the prevalence of postpartum depressive symptoms. The instruments consist of 3 parts—Part I, Part II, and Part III.

*Part I*: questions related to sociodemographic characteristics of postpartum mother.

*Part II*: questions related to postpartum depressive symptoms.

*Part III*: questions related to factors associated with depressive symptoms among postpartum mothers.

Content validity of the research instrument was established by using standard Edinburgh Postpartum Depression Scale (EPDS) [5]. It is validated and used in different cultural setting, including Nepal [9]. The research instrument was translated into Nepali language to make clear and easy for taking information from the respondents. Pretesting of tool was done on 16 postpartum mothers of ward number 29 of Bharatpur metropolitan and modification on tools done accordingly.

### 2.4. Data Analysis Procedure

The data derived from semi-structured interview were coded and entered in Epi data 3.1, and the entered data was exported into IBM SPSS 20 version. The data was analyzed and calculated according to the nature of the variable by using descriptive statistic (percentage, mean, and standard deviation) and inferential statistic (binary logistic regression and multivariate logistic regression) was used to test the association between the variables.

## 3. Result

### 3.1. Prevalence of Depressive Symptom among Postpartum Mothers

Out of 160 respondents, 27.5% of respondents had postpartum depressive symptoms and 72.5% respondent did not have depressive symptoms.

### 3.2. Association between Depressive Symptoms and Sociodemographic Factors among Postpartum Mothers

Among the study participants, most of the depressive symptoms were observed in age group > 30 years (44%). 34.8% of respondents with depressive symptoms were from nuclear family. 33.3% of participants with depressive symptoms were remarried. Various sociodemographic factors and association of depressive symptoms are summarized in Table 1.

### 3.3. Association between Depressive Symptoms and Mother-Related Factors among Postpartum Mothers

Postpartum depressive symptoms were most commonly observed among the respondents with chronic disease in family (42.1%). In the same way, depressive symptoms were observed more (42.9%) in those who did not complete 4 ANC visits in comparison with those who completed 4 ANC visit. 80% of participants with previous history of stillbirth had depressive symptoms. The various mother-related factors and their association with PPD symptoms are described in Table 2.

### 3.4. Association between Prevalence of Depressive Symptoms and Husband-Related Factors among Postpartum Mothers

Postpartum depressive symptoms were observed in respondents whose husband consumed alcohols (37%). Likewise, mental violence was observed in 52.4% of respondents with PPD symptoms. The association of PPD symptoms and husband-related factors is mentioned in Table 3.

### 3.5. Binary Logistic Regression Analysis on Prevalence of Depressive Symptoms with Selected Variables among Postpartum Mothers

The respondents whose educational level was 10 class or less were 2.78 times more likely to have depressive symptoms as compared to the respondents whose education level was >10 class (COR = 2.78, 95% CI: 1.21-6.40). In the same way, respondents whose husband's educational level was 10 class or less were 2.37 times more likely to have postpartum depressive symptoms (COR = 2.37, 95% CI: 1.04-5.41). It was found that respondent's husbands who were engaged in other occupations were 2.61 times more likely to have depressive symptoms as compared to service (COR = 2.61, 95% CI: 0.21-2.47). In the same way, respondent's husbands engaged in foreign employment were 0.73 times more likely to have depressive symptoms as compared with service (COR = 0.73, 95% CI: 1.08-11.5).

Respondents who had chronic disease in family were 2.44 times more likely to have depressive symptoms as compared to who did not have chronic disease in family (COR = 2.44, 95% CI: 1.13-5.27). Respondents who had stillbirth before had 11.5 times more likely to have depressive symptoms among postpartum mothers (COR = 2.44, 95% CI: 1.04-5.27).

The findings show that respondents who did not completed 4 ANC visits were 2.34 times more likely to have depressive symptoms than respondents who completed 4 ANC visit (COR = 2.34, 95% CI: 1.004-5.47) .

The findings also showed that the respondents whose husbands drink alcohol were 2.41 times more likely to have depressive symptoms in postpartum period as compared with those respondents whose husbands never had alcohol (COR = 2.41, 95% CI: 1.18-4.92). Similarly, those respondents who had mental violence were 3.53 times more likely to have depressive symptoms as compared to those who did not suffer from mental violence (COR = 3.53, 95% CI: 1.37–9.05).

It was found that parity of respondents was not significantly associated with depressive symptoms among postpartum mothers (Table 4).

### 3.6. Multivariate Logistic Regression Analysis of Prevalence of Depressive Symptoms with Selected Variables among Postpartum Mothers

Respondents who had educational level of ≤10 class were 3.25 times more likely to develop depressive symptoms in comparison with educational level above 10 class. In the same way, respondents who had a chronic disease in the family were 3.25 times more likely to have depressive symptoms as compared with respondents who did not have chronic disease in family.

However, other variables, education level of husband, occupation of husband, number of ANC visit, parity, alcohol consumption by husband, and mental violence were not statistically significant with depressive symptoms among postpartum mothers (Table 5).

## 4. Discussion

This community-based cross-sectional study was designed to identify the prevalence and factors associated with depressives symptoms among postpartum mothers. The prevalence of depressive symptoms in this study was 27.5%. This finding is consistent with the study conducted in Dhulikhel Hospital, Dhulikhel, Kavre and Maternity Hospital, Kathmandu [8, 10]. Likewise, the study conducted in rural tertiary care hospital of Karnataka, India, reported that 31.4% mothers had postpartum depression [11] which is consistent with the finding from Brazil [12]. However, this finding is inconsistent with the study conducted in rural population of Dhanusa district, which reported that 9.8% had postpartum depression [13]. This inconsistent result might be due to the difference in the sociodemographic factors of that particular place. Most of the participants were of young reproductive age group which is commonly observed in our country Nepal [8]. Concerning the sex of last child, 56.3% were male whereas 43.7% were females. This study is consistent with the study conducted in Vietnam [14].

The binary logistic analysis revealed depressive symptoms was 2.37 times more likely on those respondents whose husband's education level was ≤10 class as compared to husband having education level of >10 class. Similarly, a study conducted in Lalitpur, district of Nepal, also showed similar results [15].

The study revealed that the husband of respondents who were engaged in other occupation (agriculture, daily wages and other) were 2.61 times more likely to have depressive symptoms as compared with service and foreign employment and service. This study is consistent with the study conducted in India where the respondents whose husbands were engaged in farming were more likely to develop depressive symptoms [11].

Likewise, in this study, respondents who were multiparity had a 2.02 times more likely to have depressive symptoms as compared to those respondents who were primiparity. The finding of this study is also compatible with the study conducted in rural area of Dhanusha district, Nepal [13].

Respondents whose husbands consume alcohol were 2.41 times more likely to have depressive symptoms than respondents whose husband never consume alcohol. The finding of this study is in the line with study conducted in Lalitpur Nepal [15]. Similarly, the study conducted in Morang District revealed that the drinking habit of the husband was associated with depressive symptoms among postpartum mothers [16].

Participants who suffered from mental violence were 3.53 times more likely to have depressive symptoms as compared with no mental violence. A previous study reported violence as a significant cause of depressive symptoms [15]. The finding of this study showed that the respondents who have a history of stillbirth were 11.5 times more likely to develop depressive symptoms in compare with no history of stillbirth. It is supported by the study conducted in Dhanusha [13].

This study revealed that the respondents who visited ANC clinic less than 4 times during their last pregnancy were 2.34 times more likely to develop depressive symptoms in postpartum period. It may be because ANC visit provides an opportunity for the pregnant women to be aware regarding difficulties that may occur during pregnancy and postpartum period. During ANC visit, pregnant women will receive medical information over maternal psychological changes in pregnancy. So, ANC visit also works as a stress-reducing method for pregnant women to reduce risk of postpartum depression. Moreover, respondent's chronic disease, previous history of depression, multiple pregnancies, planned pregnancy, problem during pregnancy, mode of delivery, sex of last child, preferred sex of last child, gestational week, physical and sexual violence by husband, staying with husband, recent conflict with husband and family, and support from husband and family were not found to be associated with depressive symptoms.

The multivariate logistic regression analysis in this study revealed that respondents who had educational level of ≤10 years were 3.25 times more likely to develop depressive symptoms in comparison with educational level above 10 years. This finding is consistent with the study conducted in Lalitpur and Dhanusa of Nepal [13, 15].

Regarding chronic disease in the family, respondents who had a chronic disease in the family were 3.25 times more likely to have depressive symptoms in comparison with respondents who did not have chronic disease in the family. This finding is consistent with the study conducted in Oman [17] and Cameroon [18].

The limitation of this study includes the nonprobability purposive sampling technique and use of EPDS tool for the screening of depressive symptoms without any clinical diagnosis. It was cross-sectional study conducted among minimum number of postpartum women, so further analytical and interventional studies need to be conducted to find the various factors associated with postpartum depression.

## 5. Conclusion

This study was conceived to identify and understand the prevalence and factors associated with depressive symptoms among postpartum mothers. Nearly one out of four mothers is suffering from depressive symptoms. The major factors associated with postpartum depressive symptoms are education of respondents, occupation of husband, educational level of husband, alcohol taking habit of husband, chronic disease in the family, stillbirth, less than 4 antenatal visit, parity, and psychological violence.

## Figures and Tables

**Table 1:** Association between depressive symptoms and sociodemographic factors among postpartum mothers.

Sociodemographic factors	Depressive symptoms	
	Present no. (%)	Absent no. (%)
*Age*		
<20	6 (30)	14 (70)
20-29	27 (23.5)	88 (76)
≥30	11 (44)	14 (56)
*Ethnicity*		
Bramhan/Chettri	19 (24.4)	59 (75.6)
Others	25 (30.5)	57 (69.5)
*Religion*		
Hindu	34 (25.6)	99 (74.4)
Others	10 (37)	17 (63)
*Type of family*		
Nuclear family	24 (34.8)	45 (65.2)
Joint family	20 (22)	71 (78)
*Type of marriage of respondent*		
Single marriage	42 (27.3)	112 (72.7)
Remarriage	2 (33.3)	4 (66.7)
*Husband's type of marriage*		
Single marriage	38 (26.2)	107 (73.8)
Remarriage	6 (40)	9 (60)
*Total income per month (NRs)*		
≤50000	41 (29.5)	98 (70.5)
>50000	31 (4.3)	18 (85.7)
*Education level of respondents*		
≤10 class	35 (32.7)	72 (67.3)
>10 class	9 (17)	44 (83)
*Occupation of respondents*		
Housewife	41 (29.9)	96 (70.1)
Other than housewife	3 (13)	20 (87)
*Education level of husbands*		
≤10 class	35 (32.7)	72 (67.3)
>10 class	9 (17)	44 (83)
*Occupation of husbands*		
Foreign employment	15 (20.5)	58 (79.6)
Service	4(16)	21(84)
Others^℘^	25 (40.3)	37 (59.7)

^℘^Others' occupation (agriculture, daily wages, and others).

**Table 2:** Association between depressive symptoms and mother-related factors among postpartum mothers.

Mother-related factors	Depressive symptoms	
	Present no. (%)	Absent no. (%)
*Chronic disease of respondent*		
Yes	1 (33.3)	2 (66.7)
No	43 (27.4)	114 (72.6)
*Chronic disease in family*		
Yes	16 (42.1)	22 (57.9)
No	28 (23)	94 (77)
*Depression in respondent*		
Yes	1 (25)	3 (75)
No	43 (27.6)	113 (72.4)
*Depression in family*		
Yes	1 (9.1)	10 (90.9)
No	43 (28.9)	106 (71.1)
*Gravid*		
Prim	16 (22.2)	56 (77.8)
Multi	28 (31.8)	60 (68.2)
*Abortion*		
Yes	6 (23.1)	20 (7.9)
No	38 (28.4)	96 (71.6)
*Stillbirth^€^*		
Yes	4 (80)	1 (20)
No	40 (25.8)	115 (74.2)
*Multiple pregnancy*		
Singleton	44 (27.7)	115 (72.3)
Twins or triple	0 (0)	1 (100)
*Planned pregnancy*		
Yes	21 (27.3)	56 (72.7)
No	23 (27.7)	60 (72.3)
*Problem during pregnancy*		
Yes	3 (30)	7 (70)
No	41 (27.3)	109 (72.7)
*ANC visits*		
4 visits completed	32 (24.2)	100 (75.8)
4 visits not completed	12 (42.9)	16 (57.1)
*Mode of delivery*		
Vaginal	32 (24.4)	99 (75.6)
Cesarean section	12 (41.4)	17 (58.6)

**Table 3:** Association between prevalence of depressive symptoms and husband-related factors among postpartum mothers.

Husband-related factors	Depressive symptoms	
	Present no. (%)	Absent no. (%)
*Alcohol consumption*		
Yes	27 (37)	46 (63)
No	17 (19.5)	70 (80.5)
*Physical violence*		
Yes	10 (33.3)	20 (66.7)
No	34 (26.2)	96 (73.8)
*Psychological violence*		
Yes	11 (52.4)	10 (47.6)
No	33 (23.7)	106 (76.3)
*Sexual violence*		
Yes	8 (38.1)	13 (61.9)
No	36 (25.9)	103 (74.4)
*Recent conflict*		
Yes	14 (34.1)	27 (65.9)
No	30 (25.2)	89 (74.8)
*Currently lives together*		
Yes	34 (32.4)	71 (67.6)
No	10 (18.2)	45 (81.8)
*Husband support*		
Yes	31 (26.5)	86 (73.5)
No	13 (30.2)	30 (69.8)

**Table 4:** Binary logistic regression analysis on prevalence of depressive symptoms with selected variables among postpartum mothers.

Variables	Depressive symptoms		Unadjusted OR	95% CI
	Absent no. (%)	Present no. (%)		
*Respondent's education level*				
≤10 class	51 (85)	9 (15)	2.78	1.21-6.40^∗∗^
>10 class	61 (76)	39 (33)	1	
*Education level of husband*				
≤10 class	44 (83)	9 (17)	2.37	1.04-5.41^∗∗^
>10 class	72 (67.3)	35 (32.7)	1	
*Occupation of husband*				
Service	21 (84)	4 (16)	1	
Foreign employment	58 (79.6)	15 (20.5)	0.73	0.21-2.47
Others^℘^	37 (59.7)	25 (40.3)	2.61	1.22-5.59^∗∗^
*Chronic disease in family*				
Yes	22 (57.9)	16 (42.1)	2.44	1.13-5.27^∗∗^
No	94 (77)	28 (23)	1	
*ANC visits*				
4 visits completed	100 (75.8)	32 (24.2)	1	
4 visits not completed	16 (57.1)	12 (42.9)	2.34	1.00-5.41^∗∗^
*Parity*				
Primi	65 (79.3)	17 (20.7)	1	
Multi	51 (65.4)	27 (36.4)	2.02	0.99-4.11
*Stillbirth*				
Yes	1 (20)	4 (80)	11.50	1.24-105.95^∗∗^
No	115 (74.2)	40 (25.8)	1	
*Alcohol consumption by husband*				
Yes	46 (63)	27 (37)	2.41	1.18-4.92^∗∗^
No	70 (80.5)	17 (19.5)	1	
*Psychological violence*				
Yes	10 (47.6)	11 (52.4)	3.53	1.37-9.05^∗∗^
No	106 (76.3)	33 (23.7)	1	

^℘^Others' occupation (agriculture, daily wages, and others). 1 = reference group, ^∗∗^significance at 95% CI.

**Table 5:** Multivariate logistic regression analysis of prevalence of depressive symptoms with selected variables among postpartum mothers.

Variables	Unadjusted OR (95% CI)	Adjusted OR (95% CI)	*P* value
*Respondent's educational level*			
≤10 class	2.78 (1.21-6.40)	3.25 (1.10-9.58)	0.03^∗∗^
>10 class	1	1	
*Educational level of husband*			
≤10 years	2.37 (1.04-5.41)	0.81 (0.27-2.42)	0.70
>10 years	1	1	
*Occupation of husband*			
Service	1	1	
Foreign employment	0.73 (0.21-2.47)	1.06 (0.28-3.98)	0.92
Others^℘^	2.61 (1.22-5.59)	1.87 (0.77-4.56)	1.87
*Chronic disease in family*			
Yes	2.44 (1.13-5.27)	3.25 (1.29-8.16)	0.01^∗∗^
No	1	1	
*ANC visits*			
4 visits completed	1	1	
4 visit not completed	2.34 (1.00-5.41)	1.61 (0.57-4.52)	0.36
*Parity*			
Primi	1	1	
Multi	2.02 (0.99-4.11)	1.52 (0.66-3.50)	0.31
*Alcohol consumption by husband*			
Yes	2.41 (1.18-4.92)	1.77 (0.76-4.12)	0.81
No	1	1	
*Psychological violence*			
Yes	3.53 (1.37-9.05)	1.90 (0.63-5.73)	0.24
No	1	1	

^℘^Others' occupation (agriculture, daily wages, and others). 1 = reference group, ^∗∗^significance at 95% CI.

## Data Availability

All authors had full access to the data and materials. Data are available within this article. Detailed data is available from the authors upon reasonable request.

## Abbreviations

EPDS: Edinburgh Postnatal Depression Scale
PPD: Postpartum depression
ANC: Antenatal care.
